# Follow-Up of a Case of Dopamine-Mediated Yawning-Fatigue-Syndrome Responsive to Opioids, Successful Desensitization via Graded Activity Treatment

**DOI:** 10.3390/neurolint13010008

**Published:** 2021-02-25

**Authors:** Payam Dibaj, Dagmar Seeger, Jutta Gärtner, Frank Petzke

**Affiliations:** 1 Center for Rare Diseases Göttingen (ZSEG), University Medical Center Göttingen, Georg August University, 37075 Göttingen, Germany; gaertnj@med.uni-goettingen.de; 2 Department of Neurogenetics, Max-Planck-Institute for Experimental Medicine, 37075 Göttingen, Germany; 3 Center for Neurology, Asklepios Hospitals Schildautal, 38723 Seesen, Germany; 4 Pain Clinic, Center for Anesthesiology, University Medical Center Göttingen, Georg August University, 37075 Göttingen, Germany; dseeger@med.uni-goettingen.de (D.S.); frank.petzke@med.uni-goettingen.de (F.P.); 5 Department of Pediatrics and Pediatric Neurology, University Medical Center Göttingen, Georg August University, 37075 Göttingen, Germany

**Keywords:** yawning, fatigue, exercise, desensitization, graded activity

## Abstract

A 60-year-old man suffering from recurrent attacks of yawning-fatigue-syndrome, triggered by mild exercise of his right leg since a temporary lumbar disc herniation 9 years ago, was initially treated with the oral µ-opioid-receptor agonist tilidine before each bout of exercise (see Dibaj et al. 2019 JAMA Neurology 2019;77:254). During the first few months, this treatment continuously prolonged the time without exercise-triggered yawning and fatigue. In the next few months of treatment, exercise was increased in a graded manner to alleviate the yawning-fatigue-syndrome. The number of repetitions of the physical exercises was gradually increased without taking the opioid beforehand. After several months, almost the same effort level without medication could be achieved by graded activity as before under the influence of opioid medication. Graded physical activity can thus disrupt complex pathophysiological mechanisms leading to yawning and fatigue.

## 1. Introduction

We recently described the case of a 60-year-old man who had recurrent attacks of yawning followed by severe fatigue initiated by relatively mild exercise of his right leg [1]. A first pathophysiological consideration based on the putative assumption that yawning and fatigue could result from associative activation of patient’s dopaminergic systems. Application of the dopamine agonist apomorphine alone resulted in a similar attack of yawning and severe fatigue as before during the exercise of his right leg. As shown in the videos of our previous publication, it should be emphasized that the patient’s yawning attacks were always directly associated with severe fatigue, which forced the patient to stop any exercise and lie down to rest. Both mild exercise and apomorphine could similarly trigger not only the yawns, but also the associated fatigue.

Dopamine elicits yawning through activation of D3 receptors in the hypothalamic paraventricular nucleus, whereas opioid agents inhibit yawning through activation of µ-opioid receptors in the paraventricular nucleus [2,3,4]. Our patient was subjected to a treatment regime with the weak oral µ-opioid-receptor agonist tilidine (50 mg + 4 mg naloxone, slow release preparation) before each planned bout of exercise; this treatment entailed that strain of the right leg did not trigger yawning and fatigue anymore [1]. However, the purpose of this follow-up study was to desensitize the patient from the yawning-fatigue-syndrome by continuously increasing the load on the right leg and, if possible, without first taking the opioid. Behavioral therapy regimes, e.g., graded activity treatment, have been shown to reduce severe symptoms of fatigue in cancer patients [5]. Graded activity treatment can also improve the physical activity in patients with inflammatory knee and hip diseases [6].

## 2. Case Presentation

**Figure 1:**
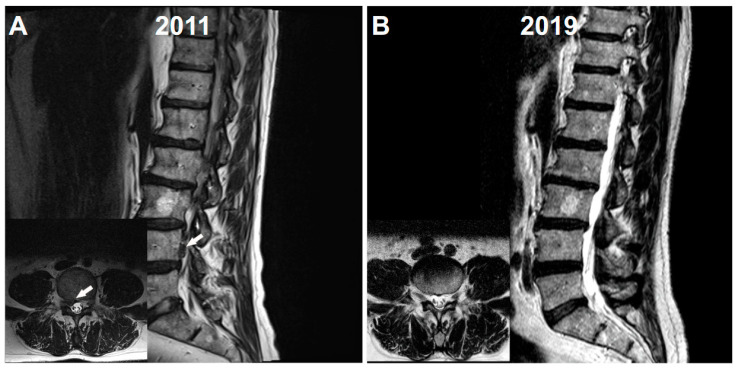
(**A**) T2 weighted MRI imaging from 2011 shows a herniation of the L4-5 disc. A cranially migrated sequestered disc fragment is marked by an arrow. The insert shows corresponding transversal MRI imaging. The herniation compresses the right L4 root (marked by an arrow). (**B**) Corresponding T2 weighted MRI sagittal and transversal (insert) images from 2019. No herniation of the L4-5 disc can be observed anymore. The right L4 root is not affected (see insert).

The yawning-fatigue attacks in the case of the 60-year-old man began a few months after a herniation of the L4-5 disc that, 9 years ago, affected the right L4 root (Figure 1A) even though sciatic pain could be reduced noticeably and relatively promptly through conservative treatment. The herniated disc was fully reabsorbed 8 years later (Figure 1B). As noted above, the patient’s yawning-fatigue-syndrome was successfully treated by the opioid tilidine. The initial aim of the therapy was a continuous increase in the load during exercise.

**Figure 2:**
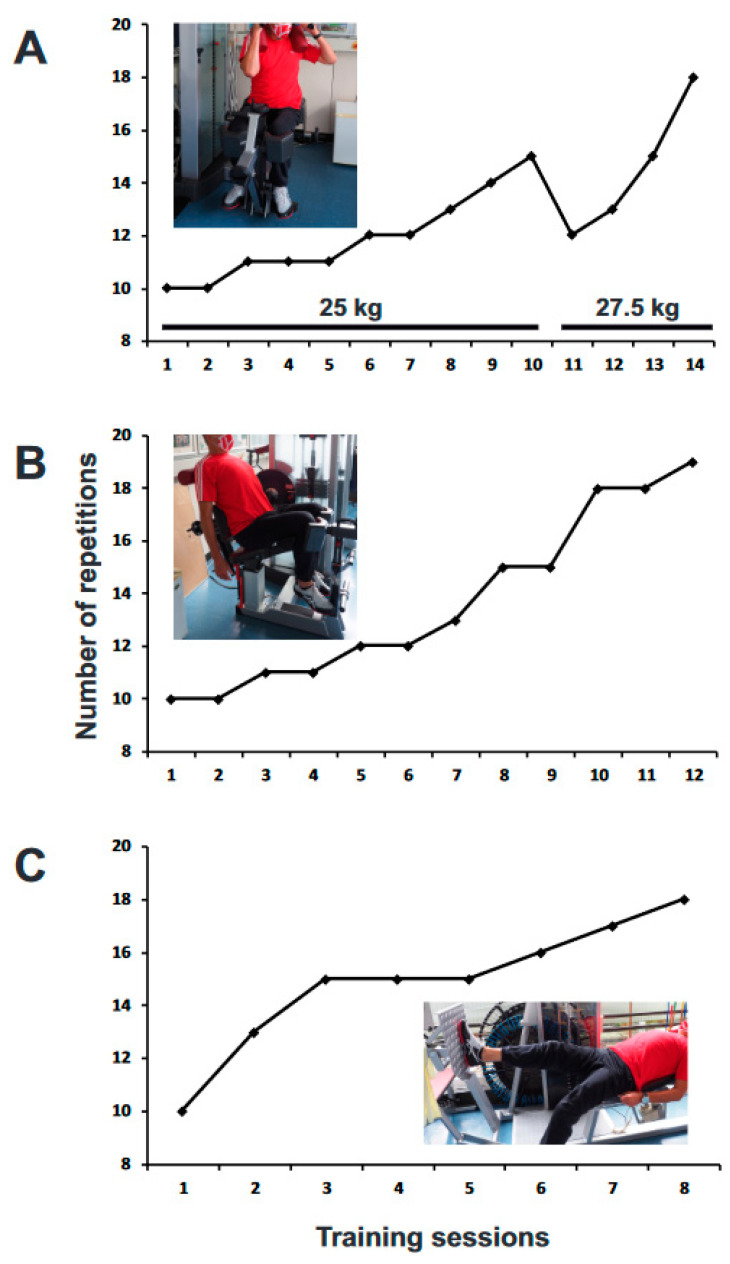
Number of repetitions of different physical exercises without attacks during treatment as a function of training session. Number of repetitions was gradually increased over the course of graded activity. Each physical exercise was performed three times per training session. The average values of the number of 3 different exercises are plotted. Each training session was performed once a week. (**A**) Rotation of the trunk. In the first 10 training sessions, a weight of 25 kg was tolerated without attacks, in the next 4 sessions a weight of 27.5 kg was tolerated. (**B**) Stretching of the trunk against a weight of 25 kg. (**C**) Leg press using only the right leg against a weight of 40 kg.

In the first 4 months of treatment, tilidine was applied 30 min before each bout of exercise. Application of the opioid increased the period of low-resistance exercise on a bicycle ergometer without attacks from half a minute to more than 10 min. Moreover, higher efforts of the trunk and extremity muscles, especially of the right leg, were possible without triggering yawning and fatigue. The patient’s graded activity program focused primarily on the load on his right leg and the adjacent trunk. During these 4 months, higher weights could be increasingly applied when rotating and stretching the trunk (see the insert images in Figure 2A,B). Leg press with the right leg was initially made only possible by taking tilidine beforehand (see the insert image in Figure 2C). Here, too, a continuous increase in the load became possible. During these exercises, attention was paid to keep the Borg rating of perceived exertion [7] at a moderate level of intensity.

The final aim of the therapy was to enable the patient to perform appropriate strains of his right leg without the need of preceding opiate administration. The therapeutic technique of graded activity [8] was subsequently used to facilitate the patient’s desensitization from the yawning-fatigue-syndrome. Thus, tilidine was not applied anymore before each bout of exercise. The patient performed, after determination of baseline quotas [8], the previously selected exercises of the trunk and extremity muscles (Figure 2). The numbers of repetitions of the physical exercises that were not interrupted by yawning-fatigue attacks gradually increased over the course of graded activity treatment. Figure 2 shows the average number of rotations (see the insert image in Figure 2A) and stretches (see the insert image in Figure 2B) of the trunk as well as the average number of leg presses with the right leg (see the insert image in Figure 2C) as a function of training session. As before, the Borg rating of perceived exertion was kept at a moderate level of intensity. Taking this into account, no attacks of yawning with following fatigue were observed in the patient. An analysis of the patient’s yawning frequency during the trials was, therefore, not necessary. The period of well-tolerated low-resistance exercise on a bicycle ergometer was similarly increased over the course of the treatment (data not shown). In all selected physical exercises, the same effort level was almost achieved by graded activity alone after 3–4 months as before, under the influence of opioid medication.

## 3. Discussion

In the present case, graded physical activity was successfully used for the treatment of a yawning-fatigue-syndrome. As before, under the influence of opioid medication, physical activity could be gradually increased. Thus, graded activity could replace the drug therapy, a rarely described change in the therapeutic regime.

Graded activity programs have been shown to be an effective therapy for a variety of patients, such as those with chronic low back pain [9]. Compared to multidisciplinary biopsychosocial rehabilitation in these patients suffering from pain, graded activity similarly reduces pain, functional disabilities and time away from work. Even for the prevention of chronic low back pain, the graded activity technique can be successfully integrated into a promising strategy in behavioral therapy [10]. Furthermore, the effect of graded activity is currently tested in an early post-surgical rehabilitation program [11]. This clinical trial will primarily include patients with chronic low back pain who are undergoing lumbar spinal fusion. As mentioned above, graded activity treatment can both reduce symptoms of fatigue in cancer [5] as well as increase physical activity in inflammatory diseases [6]. In stroke patients, graded activity training can relieve persistent fatigue after brain ischemia [12]. In this context, it must be emphasized that graded activity therapies are not always harmless in patients with chronic fatigue syndrome [13]. Caution should be exercised before proposing that any individual patient can safely increase their overall level of activity.

Significant advances in understanding the genesis of yawning have not yet fully elucidated its physiological role [14,15]. The release of dopamine and the subsequent activation of D3 receptors in the diencephalon play a key role in regulating yawning [2,3,4]. Yawning has been observed in different neurological disorders [15,16], e. g. in the prodromal phase of migraine [17,18] or together with parakinesia brachialis oscitans in stroke patients [19,20]. Even so, yawning is rarely a major feature of neurological disorders. In the present case, attacks of yawning and following severe fatigue were triggered by relatively light strain on a leg, starting 9 years ago by a prolapse of the L4-5 intervertebral disc and the resulting compression of the corresponding L4 root. Hypothalamic D3 dopamine receptors might play a key role in inducing yawning also in the case described, since the application of the dopamine agonist apomorphine alone induced similar attacks [1]. Pain caused by the disc herniation and the associated release of inflammatory or stress-related mediators such as glucocorticoids were probably not responsible for the attacks: first, sciatic pain could be reduced noticeably and relatively promptly through conservative treatment 9 years ago; second, the herniated disc was completely reabsorbed in a later lumbar MRI; third, as noted above, activation of dopamine receptors by apomorphine alone induced similar bouts of yawning and fatigue. However, the relationship between the exertion of the previously affected right leg and a putative activation of the hypothalamic dopaminergic system remains unclear. The conditioning of a sensitive dopaminergic system in the man’s diencephalon might explain the unusual connection.

Alternative mechanisms should be considered. Yawning has been shown to act as a compensatory brain cooling mechanism [21,22]. Graded activity treatment improves cardiovascular functions and yawning has thermoregulatory functions, which in the present case could in part explain why yawning disappeared with the exercises. However, some facts cannot be entirely explained by this mechanism: First, relatively mild exercise with the previously affected right leg, such as a few repetitions of low-weight presses, triggered attacks of yawning and fatigue, without straining the circulation. By contrast, the greater strain on the cardiovascular and respiratory systems elicited by the greater exertion of the other leg, trunk or both arms after long-lasting training did not trigger any attacks. Second, the patient’s graded activity program focused primarily on loading the previously affected right leg and the adjacent trunk, not on improving of cardiovascular function and endurance. Nonetheless, the influence of the latter as well as of a potential placebo effect on the success of the therapy cannot be ruled out. Third, the beneficial effect of the µ-opioid-receptor agonist piritramide was immediately effective in the patient without sufficient time for an improvement of circulatory functions [1]. Although the brain temperature was not measured directly in our previous study, the patient’s body temperature and circulatory parameters such as blood pressure and heart rate on the treadmill did not differ before and after application of the opioid. However, it cannot be ruled out that a direct suppressive effect of the relatively strong opioid did not allow yawning with its thermoregulatory effect to develop, especially since a slow increase in the load was necessary later when taking the weaker opioid tilidine.

## 4. Conclusions

In a rare case of recurrent attacks of yawning and subsequent severe fatigue, triggered by mild exercise of the right leg, graded physical activity treatment has been shown to be a suitable therapy option for decoupling yawning-fatigue from physical exercise. The precise mechanisms remain to be elucidated.
